# Growth and remodeling play opposing roles during postnatal human heart valve development

**DOI:** 10.1038/s41598-018-19777-1

**Published:** 2018-01-19

**Authors:** Pim J. A. Oomen, Maria A. Holland, Carlijn V. C. Bouten, Ellen Kuhl, Sandra Loerakker

**Affiliations:** 1 Department of Biomedical Engineering, Eindhoven University of Technology, Eindhoven, 5600MB Netherlands; 2 Institute for Complex Molecular Systems, Eindhoven University of Technology, Eindhoven, 5600MB Netherlands; 3 Department of Mechanical Engineering, Stanford University, Stanford, CA 94305 USA; 4 Department of Aerospace and Mechanical Engineering, University of Notre Dame, South Bend, IN 46556 USA; 5 Department of Bioengineering, Stanford University, Stanford, CA 94305 USA

## Abstract

Tissue growth and remodeling are known to govern mechanical homeostasis in biological tissue, but their relative contributions to homeostasis remain unclear. Here, we use mechanical models, fueled by experimental findings, to demonstrate that growth and remodeling have different effects on heart valve stretch homeostasis during physiological postnatal development. Two developmental stages were considered: early-stage (from infant to adolescent) and late-stage (from adolescent to adult) development. Our models indicated that growth and remodeling play opposing roles in preserving tissue stretch and with time. During early-stage development, excessive tissue stretch was decreased by tissue growth and increased by remodeling. In contrast, during late-stage development tissue stretch was decreased by remodeling and increased by growth. Our findings contribute to an improved understanding of native heart valve adaptation throughout life, and are highly relevant for the development of tissue-engineered heart valves.

## Introduction

Scientists have been intrigued for many centuries by the intrinsic capability of biological tissues to actively change their form and properties. While these changes are most prominent during embryonic development, most living tissues are in a continuous state of adaptation. Since the time of Galileo Galilei, it has been believed that these changes are strongly influenced by mechanical factors^1^. Now, almost four centuries later, functional tissue adaptation, and its relation with mechanics, is still only partially understood. From a biomechanical perspective, it is well accepted that growth (here defined as mass or volume change), and remodeling (here defined as property change) occur at least partly in response to changes in the tissue’s mechanical environment, in order to maintain a homeostasis^2–6^. Different mechanical constituents have been proposed to determine mechanical homeostasis across different tissue types, including stress^7–10^, strain^11–14^, and strain energy density^15–17^.

By investigating the postnatal development of native heart valves, we can increase our understanding of how growth and remodeling maintain mechanical homeostasis in heart valves throughout life. During physiological development, the environment of the aortic and pulmonary heart valves, i.e. the annulus radius^14,18^ and blood pressure^19–21^, changes significantly. As a response to these changes, tissue growth and remodeling are believed to occur to maintain mechanical homeostasis^14,22^. Tissue growth occurs through production of new extra-cellular matrix (ECM), which leads to changes in leaflet length, area, and thickness^14,18,23^. Tissue remodeling, on the other hand, has been observed through a change in ECM structure and composition^14,18,22,24–26^. The most interesting changes in the ECM properties, from a biomechanical perspective, occur in the leaflets’ collagen architecture, which is the main determinant of the mechanical behavior of heart valves. The collagen network matures with age, represented by thicker collagen fiber bundles^14,22,26^ and increasing cross-link density^18^. As a result of the remodeling of the collagen network, the stiffness of the leaflets generally increases with age^14,18,25–28^.

Recent research from our group on postnatal human native heart valves has shown relatively small differences in tissue stretch during development, and between aortic and pulmonary valves, thereby suggesting that tissue adaptation in this particular tissue occurs to maintain a stretch-driven homeostasis^14^. However, the relative roles of growth and remodeling in maintaining stretch homeostasis are still unclear. An improved knowledge of these roles is pivotal to understand the functioning of heart valves during health and disease, as adverse growth and remodeling can lead to valvular pathologies^11,12,29,30^. Moreover, growth and remodeling are of key importance for the emerging field of heart valve tissue engineering, which aims to create living heart valves that have the capability to grow and and remodel after implantation^31–36^.

During postnatal valvular development, growth and remodeling occur simultaneously^18^, which makes it challenging to experimentally deduce their relative contributions to maintaining mechanical stretch homeostasis. Therefore, numerical models can be particularly useful to study their relative roles. The past two decades, two major approaches have been used to model growth and remodeling in collagenous tissues: constrained mixture models^37^ and kinematic growth models^5,38,39^. Constrained mixture models may provide the more physiological representation of growth and remodeling. However, their parameters are hard to derive from experimental data, as they are based on the turnover of ECM components. Kinematic growth models, on the other hand, consider growth as isotropic or anisotropic volumetric changes, typically driven by a certain mechanical stimulus to reach a homeostatic target. In addition, when experimental data is available, kinematic growth could be defined in terms of known geometrical changes, rather than being driven by a mechanical stimulus. This approach only allows for prescribing homogeneous growth, as it is based on the overall geometrical changes, and is therefore not capable of releasing local stress concentrations.

**Figure 1:**
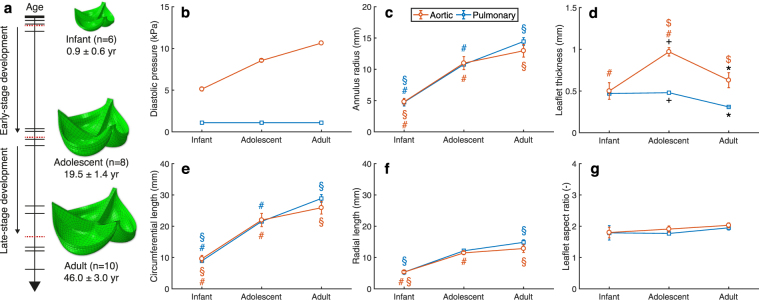
Age-dependent changes in human heart valves. (**a**) A timeline of experimental data of 24 paired human aortic and pulmonary heart valves (where each horizontal bar represents the age of one pair) were assigned to three age groups: infant, adolescent and adult (where the red dashed bars indicate the average age of each group). The age-dependent data informed the generation of a finite element model for each group. Temporal changes were considered in diastolic blood pressure^19–21^ (**b**), annulus radius (**c**), leaflet thickness (**d**), circumferential (**e**) and radial (**f**) leaflet length, and aspect ratio between the circumferential and radial length (**g**)^14,18^. Significant differences (p < 0.05) of geometrical measurements (**c**–**g**) between groups are indicated by paired symbols.

The goal of the current study was to use a numerical model fueled by experimental data to elucidate the relative roles of tissue growth and remodeling in preserving the mechanical stretch homeostasis during physiological valvular development. In these mechanical models, growth was implemented via changes in leaflet area and thickness according to finite growth theory, and microstructural remodeling via prescribing changes in the material properties. Our mechanical model was informed by hemodynamic, geometric, mechanical, and structural data of paired aortic and pulmonary human native heart valves from infant to adult origin^14,18–21^. We used a generic approach to divide age-dependent hemodynamic, geometric and structural data for aortic and pulmonary valves to three groups: infant, adolescent and adult (Fig. 1a). Between these three groups, two developmental stages were addressed: early-stage development, from infant to adolescent, and late-stage development, from adolescent to adult. During these stages, the independent influences of growth and remodeling on leaflet stretch and valve function were studied. Moreover, we systematically studied the effects of growth and remodeling on the temporal evolution of the elastic stretches during development. To this end, we varied the initial rates of growth and remodeling relative to the environmental changes and each other, and studied their influences on the temporal evolution of the elastic stretch. With this framework we show that growth and remodeling appear to play opposing roles in terms of preserving stretch homeostasis during the development of heart valves.

## Results

### Temporal changes occur in and around the human native heart valve during development

The hemodynamic, geometric and structural properties of 24 previously analyzed paired aortic (n = 12) and pulmonary (n = 12) human native heart valves^14,18^ were assigned to three age groups: infant (n = 6, average age 0.9 ± 0.6 yr), adolescent (n = 8, average age 19.5 ± 1.4 yr) and adult (n = 10, average age 46 ± 3.0 yr) (Fig. 1a). Several changes were considered between the age groups. In the valves’ environment, the aortic diastolic pressure increased with age (Fig. 1b), and the annulus radius increased significantly during early development (*p* < 0.01 for aortic and p < 0.05 for pulmonary) and only slightly during late development (Fig. 1c). Within the valves themselves, aortic leaflet thickness increased significantly (p < 0.01) during early-stage development, followed by a significant (p < 0.05) decrease during late-stage development (Fig. 1d). The adolescent (*p* < 0.001) and adult (*p* < 0.01) aortic valves were thicker than their pulmonary counterparts. The circumferential and radial leaflet length increased with age (Fig. 1e,f), while no significant differences were found between the aortic and pulmonary leaflet lengths. Finally, no significant differences were found in the circumferential-radial length ratio with age and between the two valve types (Fig. 1g).

**Figure 2:**
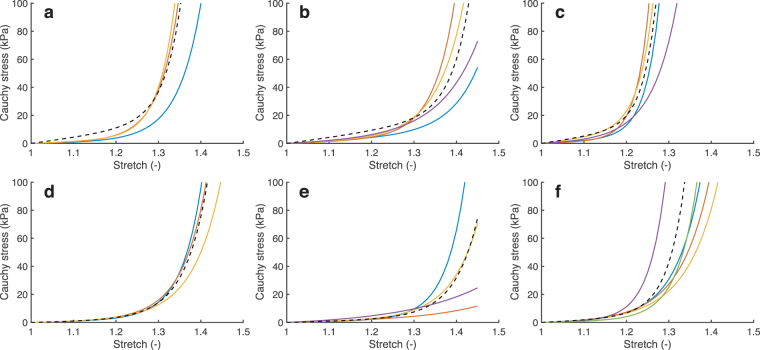
The material behavior of the aortic (**a**–**c**) and pulmonary (**d**–**f**) valves of infant (**a**,**d**), adolescent (**b**,**e**) and adult (**c**,**f**) origin was previously estimated^14^. The material behavior for each age group was estimated by fitting a fiber-reinforced hyperelastic constitutive model (dashed line) to the group’s average stress-stretch response in the circumferential (shown here) and radial direction.

**Figure 3:**
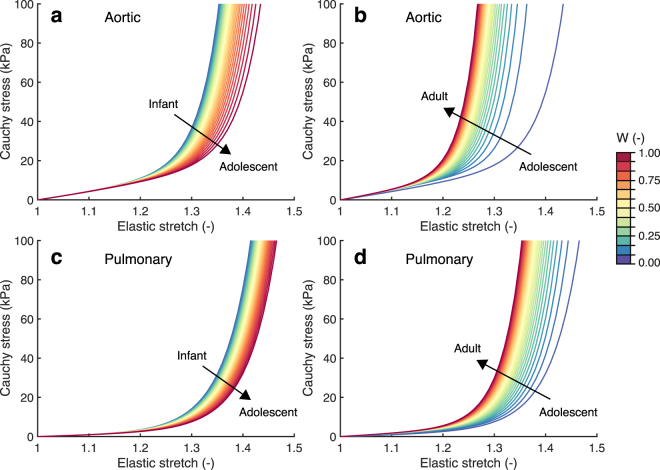
Remodeling was defined as changes in material behavior. The material properties of each age group were estimated by fitting a fiber-reinforced hyperelastic constitutive model to the group’s average stress-stretch response. The changes in material behavior during early and late-stage development were modeled as a function of a weight factor W (color legend). The circumferential stress-stretch plots indicate that the aortic (**a**) and pulmonary (**c**) leaflets became more compliant during early-stage development and became stiffer during late-stage development (**b**,**d**).

To estimate the mechanical behavior of the different age groups, a set of material parameters was determined for each age group by fitting a fiber-reinforced hyperelastic constitutive model to the group’s individual stress-stretch responses (Fig. 2). According to the stress-stretch curves, aortic leaflet stiffness decreased during early development (Fig. 3a), followed by an increase during late development (Fig. 3b). The pulmonary leaflets were more compliant than their aortic counterparts, but featured the same temporal evolution (Fig. 3c,d).

**Figure 4:**
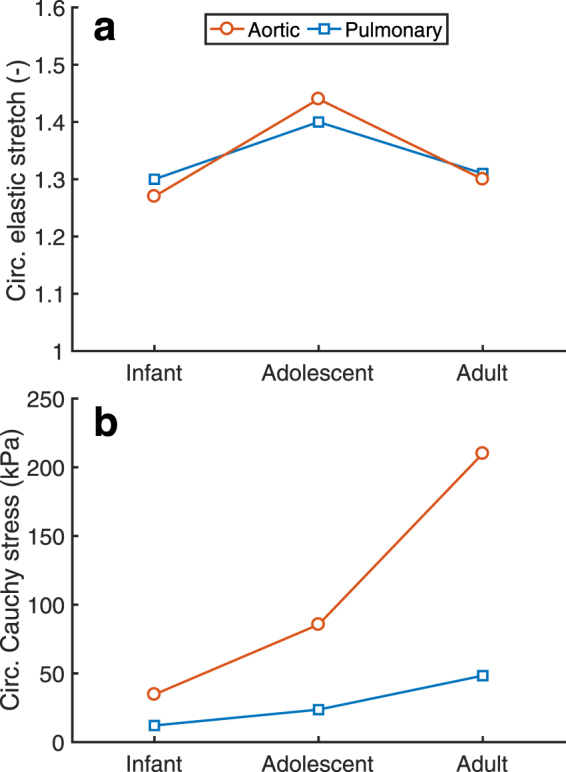
Evolution of stretch and stress with age. Age-dependent simulations indicated that the circumferential (circ.) stretch was similar with age and between the aortic and pulmonary valves (**a**), whereas the circumferential Cauchy stress was higher in the aortic valve than in the pulmonary valve and increased continuously with age (**b**).

The mechanical properties, alongside the other age-dependent data (Fig. 1), were used to generate a finite element model of each valve group in order to simulate the average mechanical state of heart valves at valve closure during diastole. The elastic stretch in the center of the belly region in the circumferential direction, which coincides with the main collagen orientation, was similar between the aortic and pulmonary valves for all age groups, and between infant and adult valves (Fig. 4a). Of note, stretch in the adolescent valves was slightly higher than in the other age groups. In contrast, the circumferential Cauchy stress in the belly region was considerably higher in the aortic valves than in the pulmonary valves, and continuously increased with age (Fig. 4b). These findings comply with our previous observations in individual valves^14^.

### During early-stage development growth reduces stretch, whereas remodeling increases stretch

Physiological valve development from the infant to the adolescent, and from the adolescent to the adult valve, was simulated by applying environmental changes (annulus radius and diastolic blood pressure) to the infant and adolescent models. With these simulations, the combined and individual influences of growth (change in leaflet length and thickness according to Fig. 1d–f) and microstructural remodeling (change in mechanical properties according to Fig. 3) after development on leaflet stretch were estimated.

**Figure 5:**
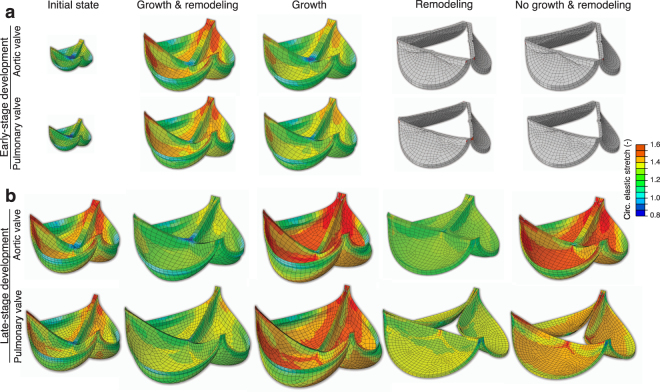
The influence of growth and/or remodeling on circumferential leaflet stretch and valve closure. Valve closure during diastole was simulated for early-stage (**a**) and late-stage (**b**) development of aortic and pulmonary heart valves and tissue stretch was assessed after applying growth and remodeling either combined or alone. Circumferential elastic leaflet stretches after growth and/or remodeling were similar in the aortic and pulmonary valves, with stretches in the early-stage valves without growth higher than 1.6. Insufficient valve closure was observed without growth after early-stage development of the aortic and pulmonary valves, and pulmonary late-stage development.

**Figure 6:**
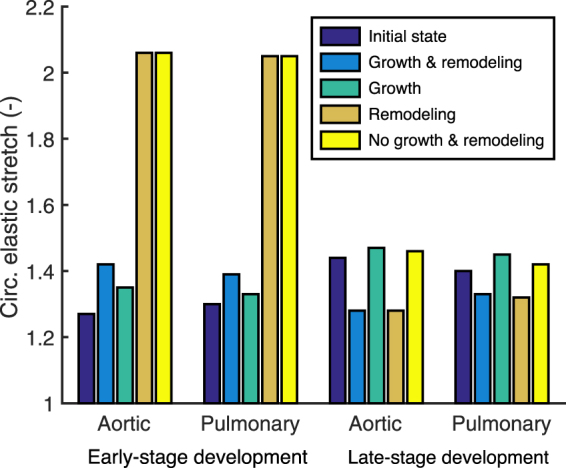
The influence of growth and/or remodeling on circumferential stretch in the belly region. For both the aortic and pulmonary valve, growth and remodeling appeared to play opposing roles on tissue stretch and with time. Growth decreased circumferential stretch during early-stage development while remodeling lead to and increase in stretch, and vice versa for late-stage development.

Growth and/or remodeling during early-stage development had a similar effect on tissue stretch in both the aortic and pulmonary valve, despite their different mechanical, geometric and environmental properties (Figs 5a and 6). Tissue growth alone reduced circumferential stretch after early-stage development towards homeostatic values. In contrast, with remodeling alone, or without growth and remodeling altogether, the stretches after early-stage development were extremely high. Interestingly, the combination of growth and remodeling also prevented excessive stretches, although the stretches were larger compared to applying growth alone, and to the initial state. The latter result coincides with the elevated stretch that we found in the adolescent valve (Fig. 4a). Incomplete valve closure was observed without leaflet growth, while remodeling was not observed to significantly influence valve closure (Fig. 5a).

### During late-stage development remodeling reduces stretch, whereas growth increases stretch

Similar to early-stage development, growth and/or remodeling during late-stage development had comparable effects on leaflet stretch between the aortic and pulmonary valve (Figs 5b and 6). Interestingly, during this stage the effects of growth and remodeling on tissue stretch were opposite compared to early development: remodeling alone reduced the circumferential stretch, while growth alone increased tissue stretch. When growth and remodeling were combined, the stretch was reduced compared to the initial state, which coincides with the lower stretch that we found in the adult valve compared to the adolescent valve (Fig. 4a). Incomplete valve closure was only observed in this stage in the pulmonary valve when no growth was applied (Fig. 5b).

### Leaflet volume changes primarily during early development

**Figure 7:**
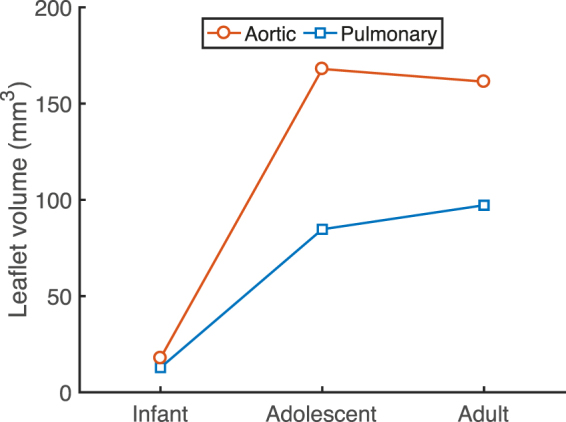
Changes in leaflet volume with age. Single leaflet volume in the models of both aortic and pulmonary valves increased dramatically during early-stage development due to tissue growth, whereas no clear change in volume was observed during late-stage development. The volume of the aortic valve was always higher than that of the pulmonary valve.

During both early and late-stage development, leaflet area was found to increase with age, while thickness increased during early-stage development and decreased during late development (Fig. 1d–f). As a consequence, the total leaflet volume of the models increased dramatically during early-stage development for both valves (Fig. 7), whereas stabilization of leaflet volume was observed during late-stage development. This suggests that the changes in leaflet geometry during late-stage development are rather due to tissue dilation than to growth.

### Importance of growth and remodeling kinetics

**Figure 8:**
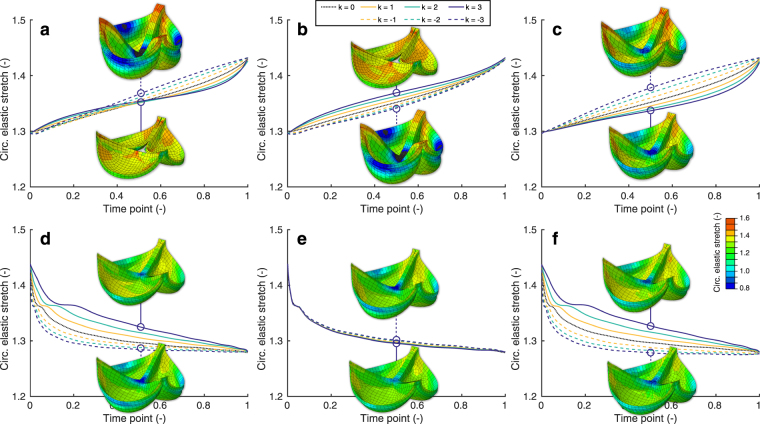
Importance of growth and remodeling kinetics. The relative rates *k* of growth (*k* = *k*_g_) and remodeling (*k* = *k*_r_) with respect to the environmental changes were altered to study their effects on the temporal stretch evolution. Three cases were investigated for early (**a**–**c**) and late-stage (**d**–**f**) development: (**a**,**d**) changing growth rate *k*_g_ and remodeling rate *k*_r_ simultaneously, (**b**,**e**) changing only the growth rate *k*_g_ while *k*_r_ = 0, and (**c**,**f**) changing only the remodeling rate *k*_r_ while *k*_g_ = 0. Growth and remodeling occurred with the same rate as the environmental changes if *k* = 0, initially slower if *k* < 0; and initially faster if *k* < 0. The three-dimensional circumferential stretch distributions are shown halfway the developmental steps at time point 0.5 for *k*_g_ = ±3 and/or *k*_r_ = ±3.

The model was used to systematically study the effects of growth and remodeling on the temporal evolution of the elastic stretches during development. To this end, we varied the initial rates of growth and remodeling relative to the environmental changes and each other, and studied their influences on the temporal evolution of the elastic stretch. Rate change parameters were introduced for both growth (*k*_g_) and remodeling (*k*_r_), which governed their initial rate change relative to the environmental changes. Overall, the aortic and pulmonary valves showed similar temporal trends in terms of stretch, so for the sake of brevity only the results for the aortic valve are shown (Fig. 8).

During early development, the stretch increased more or less linearly when growth and remodeling occurred at the same rate as the environmental changes (Fig. 8a–c). The changes in growth rate affected leaflet stretch most at the beginning of the developmental time, with a slower relative growth rate (*k*_g_ > 0) leading to increased intermediate stretches, observed at the halfway point of early development (Fig. 8b). In contrast, the changes in remodeling rate had the largest effect at the end of the development, with a faster rate (*k*_r_ < 0) leading to higher intermediate stretches (Fig. 8c). Accordingly, when both rates were changed simultaneously, growth was most most influential at the beginning, and remodeling at the end of development (Fig. 8a), with a transition taking place halfway. When growth initially occurred slower than the environmental changes, no full valve closure was observed halfway the developmental time.

Interestingly, during late-stage development, the stretch decreased in an exponential decay fashion when growth and remodeling occurred with the same rate as the environmental changes (Fig. 8d–f). The effect of different growth rates on the temporal change in stretch was negligible (Fig. 8d–e), while a relatively fast remodeling rate (*k*_r_ < 0) led to a fast decrease in intermediate stretches (Fig. 8d,f). Full valve closure was observed at all times.

## Discussion

Growth and remodeling are widely believed to occur to maintain a certain mechanical homeostasis^2–6^, with recent evidence indicating that in native heart valves this homeostasis is determined by tissue stretch^14^. In this study, we used mechanical models that were informed by experimental data to elucidate the relative roles of growth and remodeling on the preservation of tissue stretch in aortic and pulmonary human native heart valves during physiological development.

### Growth and remodeling have opposing effects on valve tissue stretch and with age

Our results indicate that growth and remodeling play opposing roles in preserving tissue stretch and with time. During early-stage development, growth preserved tissue stretch, whereas remodeling led to an increase in stretch (Figs 5a and 6). These effects occurred more rapidly when the initial growth and remodeling rates were increased (Fig. 8a,b). The decrease in tissue stretch via growth can be explained by the increase in both leaflet area and thickness (Fig. 1e,f). The increase in stretch via remodeling can be explained by the tissue softening that occurred during early-stage development (Fig. 3a,c). Changes in the material stiffness of heart valves by remodeling have previously been correlated with collagen cross-link formation^18,27^. Collagen cross-link formation is a much slower process than tissue growth, and cannot occur while ECM is being produced^27^. Therefore, we hypothesize that the tissue softening that was observed during early-stage development is a repercussion of growth, as an increase in tissue stiffness via remodeling may be impossible while ECM is still being produced.

In contrast to early-stage development, the influences of growth and remodeling on stretch appeared to be reversed during late-stage development. Our results indicate that during late-stage development, leaflet stretch is decreased by remodeling, and increased via geometrical changes (Figs 5b and 6). Interestingly, the geometric changes that occurred during this stage (Fig. 1d–f) did not result in a clear change in volume. Therefore, it can be concluded that valvular tissue growth, which we defined as change in volume, primarily occurs during early-stage development, with tissue dilation, rather than growth, occurring during late-stage development. Leaflet thinning due to dilation appears to be mostly responsible for the large deformations that were observed after late-stage development with only growth, in both the aortic and pulmonary valve. These deformations cannot be solely caused by the increase in pressure, since the diastolic pressure only increases for aortic valve, whereas it remains constant for the pulmonary valve throughout development.

Since no active growth took place during late-stage development, tissue remodeling is more likely to occur via cross-link formation. This could be responsible for tissue stiffening (Fig. 3b,d) and thus counteracts the increase in leaflet stretch that occurs via dilation, associated with the decrease in leaflet thickness. Aside from cross-link formation, leaflet calcification and collagen fiber alignment could have contributed to leaflet stiffening with ageing. Calcification occurs gradually with age in most valves, and increases local stiffness during late-stage development^40^. Collagen fiber alignment was previously found to increase during late development^14^ for both aortic and pulmonary valves, which coincides with the increased circumferential stiffness that was found in the same valves.

### Volumetric changes with age can be explained by mechanical stimuli

The presence of active growth during early-stage development, and its absence during late-stage development, can be explained by the influence of mechanical stimuli on valvular interstitial cells, which are responsible for leaflet growth and remodeling. In previous *in vitro* studies, it was shown that valvular interstitial cells become activated upon mechanical stimulation^41,42^, resulting in a myofibroblast-like phenotype that is associated with ECM production^22,43^. Clearly, larger overall changes in mechanical stimuli are provided via environmental changes during early-stage in comparison with late-stage development (Fig. 1b,c). These changes in mechanical stimuli activate the valvular cells and thus explains why in our study leaflet growth was only observed during early development. Leaflet dilation during late-stage development would then be a passive process. During the latter stage, the cells remain quiescent due to less prominent changes in mechanical stimuli, since the environmental changes are much smaller compared to early development.

In a physiological situation, changes in mechanical stimuli are primarily present during early-stage development. At an older age, cells can still become activated when mechanically stimulated by pathologies, as observed in dilated cardiomyopathy, where the ventricle and mitral valve annular radius are enlarged and cause increased leaflet stretch^12,44,45^. It has been demonstrated in adult human patients^11^ and animal models^12,30,46^ that this pathology results in significantly enlarged mitral leaflets, and that interstitial cells are activated^46^, which is in a physiological situation only the case during early development^18,22,43^. The occurrence of leaflet growth during cardiomyopathy suggests that growth can occur regardless of age, provided that the valvular interstitial cells are presented with changes in mechanical stimuli.

### Growth and remodeling feature similar temporal trends but different magnitudes in the aortic and pulmonary valves

It is interesting to note that the aortic and pulmonary valve operate under completely different mechanic al circumstances, since the aortic diastolic pressure, leaflet thickness, and leaflet stiffness are much higher than the pulmonary pressure. Still, the same homeostatic stretches (Fig. 4) and temporal effects of growth and remodeling on preserving stretch (Figs 5 and 6) were found for both valve types. Growth and remodeling appeared to occur proportionally to the environmental changes, with higher magnitudes in the aortic than in the pulmonary valves. More growth occurred in the aortic valve (Fig. 7), mainly characterized by a larger increase in thickness (Fig. 1d). The stiffness, modulated by remodeling, of the aortic valves was also higher than in their pulmonary counterparts (Fig. 3). This further supports the hypothesis that growth and remodeling occur to maintain a stretch homeostasis, for more growth and remodeling are required in the aortic valve to counteract the increasing aortic diastolic pressure, and thus to establish the same stretches as in the pulmonary valve.

### Limitations of the mechanical model

The current model features three main limitations. First, although the experimental foundation of the model is a great advantage, we were limited by data availability. We could now only consider developmental stages of rather large time spans of at least 18 years. Particularly, the temporal step between the infant and adolescent groups was relatively large, since extensive somatic changes take place during this transition. An intermediate group of around 12 years old would have been desirable, but no experimental data were available around this age.

Second, we did not implement prestretch, which is known to significantly influence tissue mechanics^47^. To the best of our knowledge, the only data on aortic and pulmonary leaflet prestretch has recently been obtained from valves from elderly patients (average age 63 yr) who underwent heart transplantation^48^. We used these novel findings in the generation of the leaflet geometry to ensure an appropriate circumferential-radial leaflet length ratio of 2 according to Fig. 1g. Yet, since it is still unknown if and how prestretch evolves with age, no actual tissue prestretch was implemented in the age-dependent growth and remodeling simulations.

Third, the growth and remodeling that we applied in the numerical model was different to traditional approaches, where tissue growth not only occurs to reach a mechanical homeostasis, but also to release any local stress and/or strain concentrations. Due to the global manner in which growth and remodeling were applied in our model, local artifacts in the stress and strain fields may have arisen.

### Conclusion

Our mechanical model, that was informed by experimental data, indicates that growth and remodeling play opposing roles in preserving tissue stretch and with time. During early-stage development, growth preserves tissue stretch, while remodeling leads to an increase in stretch, which we hypothesize is caused by tissue softening as a repercussion of growth. In contrast, the influences of growth and remodeling on stretch appeared to be reversed during late-stage development. During this stage, leaflet stretch was decreased by remodeling, and increased by volume changes that we now identified as dilation, rather than growth. The obtained understanding of the distinct roles of valvular growth and remodeling is pivotal for improving the knowledge of the functioning of native heart valves during health and disease. This is highly relevant for understanding pathologies like valvular stenosis and dilated cardiomyopathy, but also for heart valve tissue engineering, which aims to create heart valves that have the capability to grow and remodel after implantation.

## Methods

### Age-dependent data from human native heart valves

The mechanical models were informed by age-related properties that were obtained from healthy paired aortic and pulmonary native human heart valves (n = 24) that we analyzed previously^14,18^. The valves were obtained from postmortem donors from mixed gender, whose cause of death was not related to valvular disease or conditions known to precede valvular disease. These valves were mechanically and structurally uneffected, but were deemed unfit for implantations due to findings that contra-indicated implantation, for instance: positive bacteriological sampling, serological findings in the donor, or other procedural non-conformities that caused rejection of the donor (e.g. sexual risk behavior and/or risks in drug abuse).

To accommodate the biological variability between individual valves, the valves were assigned to three generic age groups: infant (n = 6, average age 0.9 ± 0.6 yr), adolescent (n = 8, average age 19.5 ± 1.4 yr) and adult (n = 10, average age 46 ± 3.0 yr). In between these groups two developmental stages were considered for both the aortic and pulmonary valve: early-stage development, from infant to adolescent, and late-stage development, from adolescent to adult (Fig. 1a). For each group, the average and standard deviation of the circumferential leaflet length *L*_c_, radial leaflet length *L*_r_, leaflet thickness *t*, and diastolic blood pressure (obtained from the literature^19–21^) were determined.

The data are presented as mean ± the standard error of the mean. Statistical differences between age groups and aortic and pulmonary valves were tested with two-way ANOVA, followed by Bonferroni post-hoc testing. GraphPad Prism (GraphPad Software, Inc., USA) was used for the analysis.

### Mechanical model of physiological heart valve development

Finite element models were generated for each age group and valve type based on the age-dependent leaflet dimensions in Abaqus FEA (Dassault Systèmes, Simulia Corp., Providence, RI). Only one leaflet was modeled due to symmetry. The contact between the leaflets was modeled by adding two contact surfaces directly adjacent to the ventricular side of the leaflet, parallel to the two free edges (Fig. 8). As all leaflets were assumed to deform similarly, there cannot be any slip between adjacent leaflets. Therefore, the contact was set to be frictionless.

**Figure 9:**
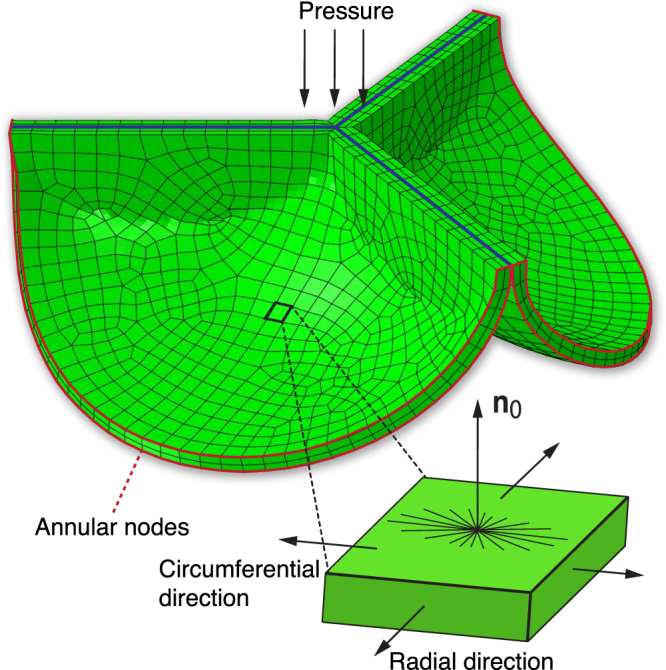
Mechanical model of heart valve development. A finite element model of a heart valve was generated for every age group based on the lower half of a spheroid. Due to symmetry, only one leaflet was modeled, and frictionless contact was modeled by adding a contact surface with friction coefficient 0 between adjacent leaflets, parallel to the free edges (blue lines). Somatic changes were applied by adjusting the diastolic blood pressure and prescribing displacement boundary conditions on the annular nodes (red lines). Leaflet stretch and stress were investigated in the highlighted element in the center of the belly region. Volumetric growth was applied in the plane spanned by the circumferential and radial direction, and in the thickness direction, given by plane normal **n**_0_. A collagen fiber network was implemented in the model which was mainly oriented in the circumferential direction.

Although we previously generated leaflet geometries based on the lower half of a spherical surface^49^, in the current study a spheroid surface with age-dependent thickness *t* was used to ensure an appropriate circumferential-radial length ratio *L*_c_/*L*_r_. The ratio *L*_c_/*L*_r_ ≈ 2.0 that we found for all valves (Fig. 1e–g) was measured *ex vivo* in excised leaflets, while it has recently been found in elderly patients who underwent heart transplantation that aortic heart valve leaflets are radially prestretched by 1.31 *in vivo*, with no evidence of circumferential prestretch^48^. Therefore, the *in vivo* leaflet length ratio should be *L*_c_/*L*_r_ ≈ 2.0/1.31 ≈ 1.53. To ensure this, the major axis of the spheroid coincided with the annular radius and was equal to half the leaflet circumferential length, and the minor principal axis length was set such that the resulting radial leaflet length (equal to a quarter of the ellipsoid minor circumference) was 1/1.53 times the circumferential length (Fig. 9).

Valvular development was simulated by first of all changing the environmental properties, i.e. annular radius and diastolic blood pressure. The former was implemented via displacement boundary conditions on the leaflet nodes at the annulus, and the latter via a change in the hydrostatic pressure that was applied to the arterial side of the leaflets (Fig. 9). Growth and remodeling were implemented and applied either combined or separately during the developmental changes, in order to assess their relative effects on preserving the leaflet stretch.

### Growth via geometrical changes

Growth was modeled as a change in volume. This was achieved kinematically according to the theory of finite growth, where the total deformation gradient tensor **F** was decomposed into an elastic contribution **F**_e_ and a growth contribution **F**_g_^38^,

1 $${\bf{F}}={{\bf{F}}}_{{\rm{e}}}\cdot {{\bf{F}}}_{{\rm{g}}}$$

Similarly, the overall volume change $J={\rm{\det }}({\bf{F}})={J}_{{\rm{e}}}{J}_{{\rm{g}}}$ was decomposed into a growth ${J}_{{\rm{g}}}=\det ({{\bf{F}}}_{{\rm{g}}})$ and elastic change ${J}_{e}={\rm{\det }}({{\bf{F}}}_{e})$. The so-called growth tensor ***F***_g_ was defined in terms of changes in circumferential leaflet length *L*_c_, radial leaflet length *L*_r_, and leaflet thickness *t*. Since the ratio between *ex vivo* circumferential and radial leaflet length remained constant during development (Fig. 1g), the relative change in circumferential (*L*_c_ + Δ*L*_c_)/*L*_c_ and radial leaflet length (*L*_r_ + Δ*L*_r_)/*L*_r_ were similar, thus defining an isotropic leaflet area change^50^ *ϑ* = (*L*_c_ + Δ*L*_c_)(*L*_r_ + Δ*L*_r_)/(*L*_c_*L*_r_). Additionally, a relative thickness change *η* = (*t* + Δ*t*)/*t* was prescribed as a uniaxial change in the direction of plane normal **n**_0_ in the reference configuration^10^. Thus we arrive at the definition of a transversely isotropic growth tensor that describes growth-induced volumetric changes in terms of area change *ϑ* and thickness change *η*,

2 $${{\bf{F}}}_{{\rm{g}}}=\sqrt{{\vartheta }}{\bf{I}}+(\eta -\sqrt{{\vartheta }}){{\bf{n}}}_{0}\otimes {{\bf{n}}}_{0}$$

### Remodeling via changes of material properties

**Figure 10:**
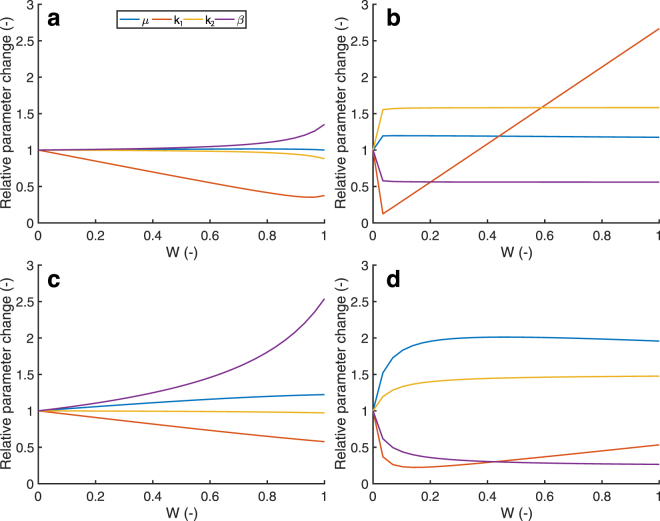
Remodeling was implemented by prescribing changes of the material properties. The four material parameters were fitted as function of a weighting factor *w* (with *w* = 0 returning the stress-stretch response before remodeling and *w* = 1 after remodeling) for aortic early (**a**) and late-stage (**b**) development, and pulmonary early (**c**) and late-stage (**d**) development.

Remodeling, e.g. stiffening due to cross-link formation, or different degrees of anisotropy due to collagen fiber reorientation leads to changes in the microstructure of valvular tissue. These microstructural changes lead to a different material response. In our model, we phenomenologically implemented microstructural remodeling by this change in material response. The (change in) material response was governed by (changes in) the material parameters of the constitutive model. These material parameters were interpolated between two developmental stages, by parameterizing them as a function of a weight factor *w* using polynomials (Fig. 10). The polynomials were fitted on the linear transition of the circumferential (*d* = circ.) and radial (*d* = rad.) Cauchy stress response:

3 $${\sigma }^{d}(w)=\mathrm{(1}-w){\sigma }_{0}^{d}+w{\sigma }_{{\rm{end}}}^{d}\quad \quad \mathrm{(0}\leqslant w\leqslant \mathrm{1)}$$

such that when *w* = 0 the Cauchy stress before remodeling ${\sigma }_{0}^{d}$ was obtained, and when *w* = 1 the stress-stretch response after remodeling ${\sigma }_{{\rm{end}}}^{d}$ was obtained (Fig. 3).

The material behavior of the individual valves was previously estimated^14^ using micro-indentation testing on a confocal microscope, in combination with inverse finite element modeling, as described before^51,52^. This method allowed for anisotropic mechanical characterization in a strain range that is physiological for heart valve leaflets. For each age group (for both aortic and pulmonary valves) the average stress-stretch response in the circumferential and radial direction was determined. The material parameters for each group were then fitted to these average stress-stretch responses (Fig. 2).

The material behavior was modeled by a hyperelastic constitutive model, where the Cauchy stress *σ* was obtained from a strain energy density function Ψ. Since growth occurs stress-free, the strain energy density function is only dependent on elastic deformation Ψ = Ψ(**F**_e_)^38^, and is therefore defined per amount of grown volume,

4 $${\boldsymbol{\sigma }}=\frac{2}{{J}_{{\rm{e}}}}{{\bf{F}}}_{{\rm{e}}}\cdot \frac{\partial {\rm{\Psi }}}{\partial {{\bf{C}}}_{{\rm{e}}}}\cdot {{\bf{F}}}_{{\rm{e}}}^{{\rm{T}}}$$

with ${{\bf{C}}}_{{\rm{e}}}={{\bf{F}}}_{{\rm{e}}}^{{\rm{T}}}\cdot {{\bf{F}}}_{{\rm{e}}}$ the elastic right Cauchy-Green deformation tensor. A fiber-reinforced material was used to model the valvular constitutive behavior, where the strain energy density function consists of an isotropic matrix part m and anisotropic fibrous part f with fiber volume fraction Φ_f_, arbitrarily chosen to be 0.5^53^,

5 $${\rm{\Psi }}=\mathrm{(1}-{{\rm{\Phi }}}_{{\rm{f}}}){{\rm{\Psi }}}_{{\rm{m}}}+{{\rm{\Phi }}}_{{\rm{f}}}{{\rm{\Psi }}}_{{\rm{f}}}$$

The constitutive behavior of the isotropic matrix part was described by a Neo-Hookean constitutive model,

6 $${{\rm{\Psi }}}_{{\rm{m}}}=\frac{\kappa }{2}{\mathrm{ln}}^{2}({J}_{{\rm{e}}})+\frac{\mu }{2}({I}_{1,{\rm{e}}}-3-2\,\mathrm{ln}({J}_{{\rm{e}}}))$$

where $\kappa =\frac{2\mu \mathrm{(1}+\nu )}{\mathrm{3(1}-2\nu )}$ is the bulk modulus, *μ* the shear modulus, and *I*_1,e_ = **C**_e_: **I** the first invariant of the elastic right Cauchy-Green deformation tensor. Quasi-incompressibility was enforced by setting the Poisson’s ratio at *ν* = 0.498. The fibrous part was modeled by an angular fiber distribution given by a periodic version of the normal probability distribution function^54,55^, with a main circumferential orientation^56^ and age-dependent dispersity *β* (Fig. 9). The direction of each fiber *i* was given by the unit vector ${{\bf{e}}}_{0}^{i}$ in the reference configuration and ${{\bf{e}}}_{{\rm{g}}}^{i}$ in the configuration after growth. Each fiber additively contributed to the total fiber strain energy density function^53^, where the strain energy density function ${{\rm{\Psi }}}_{{\rm{f}}}^{i}$ for each individual fiber *i* was given by an exponential model^14,49,53,56^,

7 $${{\rm{\Psi }}}_{{\rm{f}}}^{i}=\frac{{k}_{1}}{2{k}_{2}}\,(\exp [{k}_{2}\langle {({\lambda }_{{\rm{e}}}^{i})}^{2}-1\rangle ]-{k}_{2}\langle {({\lambda }_{{\rm{e}}}^{i})}^{2}-1\rangle -1)$$

with *k*_1_ and *k*_2_ material parameters, ${({\lambda }_{{\rm{e}}}^{i})}^{2}={{\bf{C}}}_{{\rm{e}}}:({{\bf{e}}}_{{\rm{g}}}^{i}\otimes {{\bf{e}}}_{{\rm{g}}}^{i})$ the squared elastic fiber stretch, and 〈°〉 the Macaulay brackets to enforce that the fibers only resist tension.

### Kinetics of growth and remodeling

**Figure 11:**
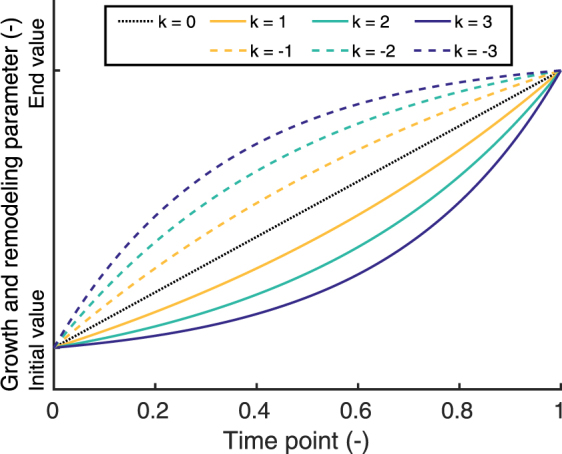
Variations in the kinetics of growth remodeling. The rate change of growth and remodeling relative to the environmental changes was investigated via a time-dependent exponential evolution of the growth and remodeling parameters. The exponential behavior was governed by rate parameter *k* (*k*_g_ for growth, *k*_r_ for remodeling), where if *k* < 0 growth and remodeling initially occurred faster, and *k* > 0 initially occurred slower compared to the environmental changes.

The environmental changes were applied as a linear function of developmental time *τ* ($0\leqslant \tau \leqslant 1$). The importance of the kinetics of growth and remodeling were investigated by defining the growth (*ϑ* and *η*) and remodeling (*w*) parameters as a function of *τ* and adjusting their rate change in an exponential fashion relative to the environmental changes, governed by rate change parameters *k*_g_ (growth rate) and *k*_r_ (remodeling rate),

8 $${\vartheta }(\tau )=({{\vartheta }}_{{\rm{end}}}-\mathrm{1)}\frac{{e}^{{k}_{{\rm{g}}}\tau }-1}{{e}^{{k}_{{\rm{g}}}}-1}+1$$

9 $$\eta (\tau )=({\eta }_{{\rm{end}}}-\mathrm{1)}\frac{{e}^{{k}_{{\rm{g}}}\tau }-1}{{e}^{{k}_{{\rm{g}}}}-1}+1$$

10 $$w(\tau )={w}_{{\rm{end}}}\frac{{e}^{{k}_{{\rm{r}}}\tau }-1}{{e}^{{k}_{{\rm{r}}}}-1}$$

where *ϑ*_end_, *η*_end_ and *w*_end_ were the parameter values at the end of the developmental stage. Note that if *k*_g_ → 0 and *k*_r_ → 0 growth and remodeling occur linearly with time (and at an equal rate to the environmental changes), if *k*_g_ < 0 and *k*_r_ < 0 growth and remodeling initially occur faster, and if *k*_g_ > 0 and *k*_r_ > 0 growth and remodeling initially occur slower compared to the environmental changes (Fig. 11).

### The relative influences of growth and remodeling

A total of six models (aortic and pulmonary infant, adolescent and adult) were prepared to study the homeostatic stretches and stress. Subsequently, early development was simulated by applying environmental changes to the aortic and pulmonary infant valves, whereas for late development these changes were applied to the adolescent valves. The relative influence of growth and remodeling on leaflet stretch was investigated by applying growth and remodeling combined, only growth, only remodeling, and no growth and remodeling. For all simulations, leaflet stretch in the middle of the belly region (Fig. 9) was investigated. The importance of the kinetics of growth and remodeling relative to the environmental changes were investigated by applying combined growth and remodeling with different rates, using three cases: changing only the growth rate (*k*_g_ = ±1, ±2, ±3 while *k*_r_ = 0), changing only the remodeling rate (*k*_r_ = ±1, ±2, ±3 while *k*_g_ = 0), and changing both rates simultaneously (*k*_g_ = *k*_r_ = ±1, ±2, ±3). For all cases, the temporal changes in valve coaptation and leaflet stretch were investigated.

### Data availability

The datasets generated during and/or analyzed during the current study are available from the corresponding author on reasonable request.
